# The Validation of the Speech, Spatial and Qualities of Hearing Scale SSQ12 for Native Romanian Speakers with and without Hearing Impairment

**DOI:** 10.3390/jpm14010090

**Published:** 2024-01-13

**Authors:** Luminita Radulescu, Oana Astefanei, Roxana Serban, Sebastian Cozma, Corina Butnaru, Cristian Martu

**Affiliations:** 1 Doctoral School, “Grigore T Popa” University of Medicine and Pharmacy, 700115 Iasi, Romania; luminita.radulescu@umfiasi.ro (L.R.); sebastian.cozma@umfiasi.ro (S.C.); 2 ENT Clinic Department, “Grigore T Popa” University of Medicine and Pharmacy, 700115 Iasi, Romania; roxana-n-serban@umfiasi.ro (R.S.); butnaru.corina@umfiasi.ro (C.B.); martu.cristian@umfiasi.ro (C.M.)

**Keywords:** asymmetric deafness, burden of hearing loss, hearing rehabilitation surgery, questionnaire, SSQ12

## Abstract

Background: The perceived impact of hearing loss varies considerably among those affected due to the heterogeneous types of hearing loss, their diverse etiologies, and the different rehabilitation possibilities. Therefore, assessing listening skills in a daily context using questionnaires is essential. This study aimed to investigate the validity and reliability of the adapted version of the Speech, Spatial and Qualities of Hearing Scale 12 (SSQ12) in the Romanian language. Materials and Methods: The SSQ12 is a 12-item self-reporting questionnaire that assesses a range of everyday listening situations. The internal consistency, test–retest reliability, and validity of the r-SSQ12 questionnaire resulting from the adaptation of the original scale were investigated. Results: The responses of 183 subjects aged between 11 and 79 years were evaluated. In total, 121 subjects had hearing loss (19 adolescents), and 62 subjects had normal hearing (11 adolescents). Significant differences were observed in the means of the overall score and for individual items between normal-hearing subjects and subjects with hearing loss. The SSQ12 had high internal consistency (Cronbach’s alpha = 0.97), and the test–retest scores were highly correlated. Conclusions: The SSQ12 scale can be used to investigate the self-reporting of hearing quality in both general populations to identify hearing disorders and populations with hearing loss.

## 1. Introduction

Hearing includes all the peripheral and central functional components of sound reception and analytic processing [1]. A World Health Organization (WHO) report revealed that by 2050, nearly 2.5 billion people will be living with some degree of hearing loss; of these, at least 700 million will require rehabilitation services [2]. The burden of hearing impairment is felt at both the institutional level (governments, WHO, and professional associations) and the individual level, where it affects the specific domain of hearing as well as the general domain of quality of life. The factors that influence the hearing trajectory across a person’s life span are genetic characteristics, health conditions or disease, behavioral factors, and environmental factors [2]. HL (hearing loss) can be present at birth (“congenital HL”), or appear sometime later in life (“acquired or delayed-onset HL”) [3]. The importance of screening children for syndromic or non-syndromic hearing loss has been demonstrated through the success of the available rehabilitation tools [3]. Screening for the common sequelae of hearing loss (e.g., communication difficulties, social isolation, mood, and quality of life) is important, even in those adults with long-term hearing loss, as needs and challenges can change over time [1].

Hearing capacity is commonly measured using pure tone audiometry and is classified based on audiometric hearing thresholds. Hearing capacity refers to the ability to perceive sounds and is commonly measured through pure tone audiometry (PTA), which is considered the gold-standard test for assessment [1]. A long audiological follow-up is of paramount importance to identify hearing threshold deteriorations early and to ensure prompt treatment with hearing aids or cochlear implants [1,3].

Tonal and speech audiometry are essential for diagnosing hearing pathologies, but they provide limited information about people’s functional hearing in everyday situations. Sensory diseases, including those associated with hearing loss, are considered to be among the most common conditions that cause chronic disability [4]. 

The term “hearing disability” includes all the problems or difficulties faced by those with hearing loss when carrying out various activities or in everyday situations, including all the impairments, limitations, and restrictions they experience (physical, social, or attitudinal) [5]. The impact of a hearing impairment depends on both the person’s clinical profile and contextual factors, such as communication needs, environmental factors, and access to rehabilitation. PROMs (patient-reported outcomes as perceived by the patient) can be considered more comprehensive than measures based only on clinical performance outcomes (objective or subjective, behavioral), in that they assess not only the functional aspects of hearing but also the socioemotional consequences related to hearing [6]. A person’s reporting of their diminished sensory capacities (in this case auditory) and perceived disability and the effectiveness of an intervention aimed at rehabilitating these capacities can be measured for the generic or specific domains of quality of life through QoL instruments [5,6,7].

The analysis and interpretation of the auditory scene are important for effective communication and a good quality of life. The concept of “auditory scene analysis” was introduced by Albert Bregman in 1990 and discussed extensively in “The Auditory System at the Cocktail Party” [8]. Hearing includes the task of recovering the coherent signals from the ensemble of all the superimposed sounds in an auditory environment, consisting of directional, distance, and motion components. Even people with mild-to-moderate hearing loss have reported that their inability to segregate multiple talkers or to understand speech in a noisy background is one of their greatest disabilities [9,10].

The Speech, Spatial and Qualities of Hearing Scale SSQ was developed to quantify the degree of disability faced by a person with impaired hearing. The abilities explored through this tool, including the ability to separate sounds, identify their spatial provenance, and understand and process speech, rely significantly on spatial hearing [9,10]. 

Many of the items on this questionnaire can be found in other hearing aid outcome-assessment tools, such as the APHAB (Abbreviated Profile of Hearing Aid Benefit) proposed by Cox and Alexander in 1995. The original questionnaire consisted of 49 items grouped into three subscales: (1) speech (e.g., speech in noise, speech in speech), (2) spatial (e.g., sound localization), and (3) other qualities of hearing (e.g., clarity or listening effort).

Cañete (2023) [11] considered the short forms available in various languages with 5, 12, 15, and 19 items (Demeester et al., 2012 [12]; Kiessling et al., 2011 [13]; Moulin et al., 2019 [14]; Noble et al., 2013 [15]); some were designed for parents, children, and teachers, and others were designed to investigate the benefits of hearing aids (SSQ-B) and compare devices (SSQ-C) (Jensen et al., 2009 [16]). The scale analysis was performed at the global level, at the level of each of the three subdomains, and at the level of the ten pragmatic subscales, and it also proposed, in addition to the three subdomains, a fourth subdomain of listening effort or fatigue. It is used in various populations: people with hearing, considered normal, without hearing loss; in different age groups; and in people with unilateral or bilateral age-related hearing loss, exposed to noise or with various otic pathologies. 

The scale with 12 items, the SSQ12A, was proposed at a conference in 2009 [16] and involved representatives from three centers (Eriksholm, MRC Institute of Hearing Research Scottish Section, and the University of New England) to assess hearing loss in the context of clinical research and rehabilitation. The decision was made that each center would independently nominate twelve items judged to be of value in a clinical context, given their experience in the use of the SSQ. In 2013, a formula was provided for converting scores between the full (SSQ49) and abbreviated (SSQ12) versions. The validity, reliability, and sensitivity of the abbreviated version are considered adequate compared to those of the full version [17,18,19]. Although the full scale of 49 items (SSQ49) is useful in this form, an abbreviated SSQ12 variant has value for rapid or routine assessments, before and after clinical treatment to manage hearing disabilities. Practical guidance for using the SSQ12 is provided by Cañete (2023) in The 12-item Speech, Spatial and Qualities of Hearing Scale questionnaire: administration suggestions and guidance [11]. There are two other SSQ12 versions available: the SSQ12-B for evaluation before and after auditory rehabilitation intervention and the SSQ12-C for comparison between the devices used. The SSQ12 can be considered a potential time-effective self-assessment of auditory function in cochlear implant (CI) recipients in their everyday routine. The SSQ12 assesses three domains of hearing with a scoring system from 0 to 10 for each question. The inability to perform the task described in each scenario is scored as 0, with 10 being the maximum if the subject rates their hearing as perfect. The domain of hearing disability is analyzed using SSQ12 in 3 subscales or in 10 pragmatic subscales:

1. The speech subscale: speech in noise (item 1 and 4), multiple speech streams (item 2 and 5), and speech in speech (item 3).
2. The spatial subscale: sound localization, sound distance, and movement (item 6, 7, and 8).
3. The Qualities of Hearing subscale: segregation, identification, naturalness, and listening effort (item 9, 10, 11, and 12).

The first section covers several realistic speech contexts with varying degrees of difficulty and different types of background noise: competing sounds, the ability to see other speakers, and other speakers engaged in a conversation; the second section addresses three components of spatial hearing—direction, distance, and movement; the third section addresses general qualities of hearing [9,10,15].

The present study aims to adapt the different SSQ12 versions (the SSQ12A, SSQ12B, and SSQC) for Romanian speakers. Cultural adaptation of the SSQ12 is also necessary for non-English speaking populations in order to also capture auditory experiences coherently and compare the effectiveness of hearing rehabilitation interventions. Documenting scientific evidence and the country’s experiences is the final goal of this approach. Considering the questionnaire’s effectiveness, validated through the assessment of hearing loss and the benefits of various rehabilitation interventions, we consider this step appropriate. The instrument in the Romanian language is developed to be used in clinical and research protocols for hearing rehabilitation with implantable devices for people with various hearing loss (especially asymmetric loss). The final subjects addressed by the SSQ have to perform many other tasks during clinical protocols (tone and speech audiogram, localization tasks, and device-fitting sessions), spending a lot of time in the clinic during the control sessions. The population the questionnaire is addressed to includes adults and adolescents over 11 years of age.

We chose to use the short version (SSQ12), guided by both the literature review and a short initial experiment with the SSQ49 with regular patients of our clinic. They failed to finalize the task of completing the questionnaire, ignoring some questions in the field of “hearing quality”, and reported that the time required for completion is “quite” long.

## 2. Materials and Methods 

### 2.1. Cultural Adaptation Procedure SSQ12

We followed the recommendations of the International College of Rehabilitation Audiology (ICRA) for the cultural adaptation procedure. ICRA is affiliated with the International Society of Audiology (ISA, http://isa-audiology.org/ accessed on 4 January 2023). The degree of urbanization, population density, recreational activities, family type, and noise level (Hall et al.) are comparable to that in any European country, which is why there are no particular problems of cultural equivalence. The Romanian language is spoken by approximately 24 million people in Romania and by a significant numerical population in the Republic of Moldova. Native speakers are relatively evenly distributed throughout the territory.

The Romanian language, the official language in Romania, is a unitary language. It originated from Vulgar Latin during the 5th and 6th centuries. There are no distinct dialects but only oral local and regional accents of the different geographical areas, with no importance to written texts. This means that the entire native population uses the written language manifested by the Romanian Academy. Because of the unitary character of the Romanian language, we decided on a standard linguistic approach without regionalisms (vocabulary, words, grammatical structures, or expressions familiar only to speakers from a certain geographical area), archaisms (expressions that are no longer part of the usual background of the majority of the active population), and neologisms (although they are part of the language of the active population, they have been excluded to address speakers of all ages).

The SSQ12 was translated according to the universal principles of the cross-cultural adaptation of instruments based on patient reports using the translation–back-translation method [20]. The authors (William Noble; Michael Akeroyd) were informed about the intention to validate the questionnaire in the Romanian language, as no validation translation existed. After this stage, the concepts corresponding to each item were defined and provided to the two people responsible for the translation.

Two practicing clinicians with experience in the field of hearing loss, who are native Romanian speakers with advanced knowledge of English, performed the translation of the instructions, the 12 questions, answers, and the attached questionnaire with 12 questions on self-esteem, disability, anchors, and additional answers for variants B and C into the target language. 

The items that required a reconciliation session between the translators were items 5 (“You are with a group and the conversation switches from one person to another. Can you easily follow the conversation without missing the start of what each new speaker is saying?”) and 9 (“When you hear more than one sound at a time, do you have the impression that it seems like a single jumbled sound?”).

Speech understanding is always better when we can turn to observe the speaker. Turning to the sound source allows better access to visual cues (speech reading) and improves the volume of the signal relative to the background noise. Quickly localizing a speaker in a group makes it easier for us to understand speech and listen attentively. In the scenario described by the question Q5, the flow of speech is fast, so the cues provided by the return to the source are not always available. 

Even though the concept of the segregation of sounds described in the scenario of Q9 is easy to understand, the word-to-word translation did not return satisfactory variants. 

The two versions were reconciled through consensus between the two translators or, for items 5 and 9, by appointing a responsible person not involved in the translation process who conducted a pilot test (debriefing) with 10 Romanian speakers aged between 15 and 75, in both the clinic and the family environment. Their suggestions referred to avoiding repetitions. The solutions were presented to the two translators, who produced a final version for back translation.

A native Romanian speaker, an experienced user of the English language who did not know the original version, performed the back translation. The back translation was subjected to verification and comparison with the original English version by a professional translator, Professor of English at the University of Medicine and Pharmacy Iași, based on an evaluation of the correspondence marked from A to E, with A and B representing scores considered acceptable without having to restart the translation process. Following the cultural validation methodology, variants were created in the target language marked with “B” (for Q5 and Q9) and “A” for the rest.

The version in the target language was submitted to a consensus session in a multidisciplinary team (a project coordinator, two hearing implant specialists, and an English language teacher). Considering that the use of self-assessment is not a routine practice for the end user (a native Romanian speaker), the clarity of the instructions, the compatibility of the answer grid with the habits and expectations of a hearing device user, and the degree of acceptance and compliance with the questionnaire were re-evaluated. The final Romanian version underwent a final quality check. The questionnaire version for the group aged 11–19 years was identical to the one for adults (Supplementary Materials). 

### 2.2. Subjects

The subjects were both volunteers with normal hearing and volunteers with hearing loss treated at the Clinical Rehabilitation Hospital in Iasi, Romania. 

The protocol for the selection of the study group included the following: the identification of potential subjects, an overview of the study methodology, an interview for data collection (age, sex, otic history, or complaints), signing the informed consent form (for subjects over 18 years old or for legal representative of subjects aged 11–18 years old), otoscopy, tympanometry test, tone audiogram, application of SSQA, SSQB, and SSQC questionnaires.

Inclusion criteria of the group with normal hearing included the following:

- PTA4 better than 25 dB in both ears;
- Self-declared good or very good hearing;
- Native Romanian speakers aged over 11 years;
- Normal otoscopic examination.

Exclusion criteria of the group with normal hearing included the following:

- History of noise exposure or chronic otological history;
- Acute otic symptoms and/or serous otitis of less than three months, chronic otitis media, acute otitis, external otitis, and acute mastoiditis;
- Illiterate people.

Inclusion criteria for the group with hearing loss include the following:

- PTA4 over 25 dB HL in at least one ear;
- Native Romanian speakers aged over 11 years.

Exclusion criteria for the group with hearing loss include the following:

- Acute otic symptoms or/and serous otitis of less than three months, acute otitis media, otitis externa, and acute mastoiditis;
- Illiterate people;
- An MMSE greater than 25 in patients who received CI or BCI or active UM prosthesis.

Exclusion was confirmed by an ENT clinician who performed the otoscopic examination and tympanometry test.

The subjects underwent a tonal audiogram in a soundproof room (acoustically treated walls and floor) used in studies initiated in our clinic to document hearing rehabilitation with different implantable devices. The device used for noise measurement [21] was a PCE-322A sound pressure level meter with a measurement window of 30 dB to 130 dB and a resolution of 0.1 dB. An automatic calibrated audiometer (equinox/affinity suite Interaccoustic) with a frequency range from 125 Hz to 8000 Hz was used. Over-the-ear headphones (TDH-39) and a bone vibrator were used as transducers. Threshold search audiometry determines the softest sound a patient can hear at each frequency 50 percent of the time. This testing requires more time and expertise than those required for screening audiometry. The American Speech-Language-Hearing Association has a recommended procedure for pure-tone threshold search tests, known as the modified Hughson–Westlake method. Testing begins with the ear in which the patient perceives themselves to have better hearing. The tester presents a pure tone at a clearly audible level. After the patient responds to the pure tone signal, the tester decreases the intensity by 10 dB and presents the tone again. If the patient responds to this tone, a “down 10” pattern is employed, with the tester decreasing the intensity of the tone by 10 dB and presenting a tone until the patient no longer responds. The tester then increases the tone intensity by 5 dB until the patient responds. Continuous pure tone signals were used. Audiometric data were reported according to recommendations from the American Speech-Language-Hearing Association [1]. 

Average PTA (PTA4) was calculated using the formula (500 Hz + 1000 Hz + 2000 Hz + 4000 Hz)/4. Any hearing loss was defined as PTA0.5–4 kHz > 25 dB [5].

The subjects completed the r-SSQ12 questionnaire in the paper-and-pencil version. All subjects with normal hearing and subjects with hearing loss who required hearing aid intervention received the r-SSQ12-A. The r-SSQ12-B for assessment before and after surgery was applied to subjects who received hearing rehabilitation surgery at least 6 months before testing and who had not previously used another hearing aid. The questionnaire r-SSQ12-C was applied to subjects who received a hearing implant or a conventional hearing aid for at least 6 months and had previously used a hearing aid. Subjects had the opportunity to ask questions to the staff who administered the questionnaires.

Thirty-eight subjects, all with hearing loss and over 19 years old, completed the formular one more time at follow-up checks (between 4 and 10 weeks) for the r-SSQ12-A for retesting.

The degree of hearing loss was considered as follows:

- Normal hearing: PTA4 better than 25 dB in both ears;
- Mild and moderate hearing loss: PTA4 between 25 dB and 70 dB HL in at least one ear;
- Severe or profound hearing loss: PTA4 greater than or equal to 70 dB HL in at least one ear.

The degree of symmetry in hearing loss was defined as follows: UHL (unilateral hearing loss with one normal ear), AHL (asymmetric hearing loss, severe or profound hearing loss in one ear, and a difference of at least 15 dB HL between ears), BHL (symmetrical bilateral hearing loss regardless of degree).

Statistical analysis was performed using the Statistical Package for the Social Sciences (SPSS) version 23.0. The skewness test and the Kolmogorov–Smirnov test were used to evaluate the hypothesis of normality of the data. To verify the statistical significance of the difference between groups, a univariate ANOVA analysis with Bonferroni correction, preceded by Levene’s test, was performed. The null hypothesis was that the means did not differ between groups. A paired sample test for testing test–retest reliability was confirmed using reliability statistics for absolute agreement. Cronbach’s alpha, Guttman’s index, and item–total correlation for SSQ12 were calculated to test internal consistency. The significance level was defined as 0.05 (5%).

## 3. Results

### 3.1. Subject Group Structure

In total, 183 subjects aged between 11 and 79 years, with a mean of 41.46 years ± 17.92 and median of 45 relatively close to the mean value, completed the r-SSQ12A questionnaires. In total, 121 subjects had hearing loss (19 adolescents), and 62 had normal hearing (11 adolescents). The age series was homogeneous. The 50–59 and 40–49 age groups were most represented among all respondents, followed by the 11–19 age group. The median age of 45 years combined with a weight of 50.3% above this was chosen as the threshold for further significance tests. The r-SSQ12-B was completed by 28 subjects who had received a first rehabilitation device and the r-SSQ12-C by 21 subjects who had changed from a hearing aid to a CI (adults only).

Regardless of the degree of hearing loss, the female gender predominated: 64.1% of cases with severe or profound loss and 62.8% of those with moderate loss (*p* = 0.985) were female. Audiometric characteristics are reported as PTA4 in Table 1.

Among the 41 subjects aged 50–59 years, 23 had severe or profound hearing loss; of the 30 subjects aged 11–19 years, 13 had severe or profound hearing loss (*p* = 0.001) (Table 2). Of all respondents, 47.5% had sensorineural hearing loss. Among the 23 respondents with conductive hearing loss, 56.5% were female (*p* = 0.013) and 60.9% were under 45 years of age (*p* = 0.005). Among the 87 respondents with sensorineural hearing loss, 60.9% were female (*p* = 0.013) and 60.9% were over 45 years old (*p* = 0.005). The 11 subjects with mixed hearing loss were female (*p* = 0.013) and 72.7% were over 45 years old (*p* = 0.005).

Among the 47 cases with UHL, 55.3% were female (*p* = 0.336) and 59.6% were under 45 years of age (*p* = 0.001). Among the 11 cases with AHL, 81.8% were female (*p* = 0.336) and 63.6% were over 45 years old (*p* = 0.001). Among the 63 cases with BHL, 66.7% were female (*p* = 0.336) and 69.8% were over 45 years old (*p* = 0.001).

Among the 47 cases with UHL, 70.2% had severe or profound hearing loss, and all 11 cases with AHL had severe hearing loss; among the 63 cases with BHL, 54% had severe or profound hearing loss.

### 3.2. r-SSQ12 Total Score Scale

There were four cases for Q2 r-SSQ12-A, two for Q4 r-SSQ12-A and one for Q5 r-SSQ12-A where subjects filled in “not applicable” for the condition at the time of r-SSQ12A application (bilateral profound hearing loss, ineffectively provided with conventional prosthesis, and CI candidates). Responses were excluded from analyses of overall scores.

The total mean r-SSQ12-A score was 6.53 ± 2.75; the mean score was 9.14 ± 0.62 for subjects with normal hearing and 5.29± 2.29 for subjects with hearing loss. For the adolescent group (11 with normal hearing and 19 with hearing loss), the mean r-SSQ12-A score was 9.42 ± 0.41 for subjects with normal hearing and 5.65 ± 2.12 (1.33–9.08) for subjects with hearing loss. The median values were close to the mean values, and the results of the skewness test of >−2 suggest that the series of values of the total score and the scores on various groups were homogeneous. This result was also reconfirmed using the Kolmogorov–Smirnov test (total score). 

**Figure 1:**
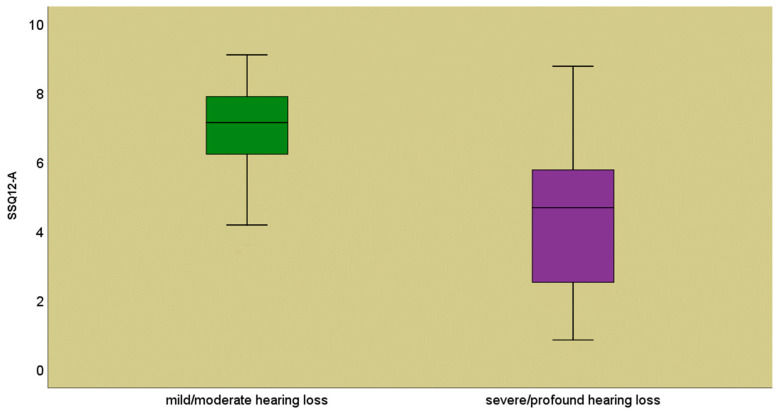
Mean values of the r-SSQ12-A score according to the degree of hearing loss.

Depending on the degree of hearing loss, the lowest mean values of the total r-SSQ12-A score were observed in the group with severe or profound hearing loss (4.14 ± 2.22) compared to the group with moderate hearing loss (6.9 ± 1.3), *p* = 0.001 (Figure 1). 

**Figure 2:**
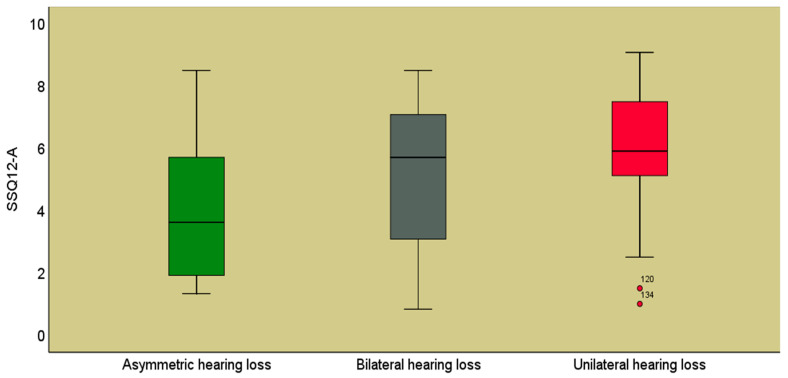
Mean values of the r-SSQ12-A score according to the symmetry of hearing loss.

The highest average values of the total r-SSQ12-A score were recorded in the UHL group (6.03 ± 2.02) and the lowest were recorded in the AHL group (4.22 ± 2.43), *p* = 0.001, between these groups (Figure 2).

**Figure 3:**
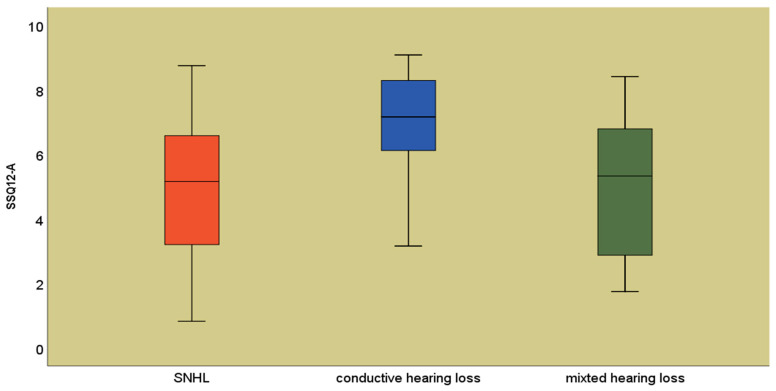
Mean values of the r-SSQ12-A score according to the type of hearing loss.

Depending on the type of hearing loss, the highest mean values of the total r-SSQ12-A score were observed for conductive hearing loss (7.08 ± 1.72), and the lowest were observed for sensorineural hearing loss (4.69 ± 2.3) (Figure 3). 

The scores for the mean total score of the SSQ12A differ in statistical significance between the groups formed according to the type of hearing loss (Table 3).

### 3.3. Scores on Items

The scores on each question differ in statistical significance between the groups formed according to the degree of symmetry in the hearing loss (Table 4).

Retesting was performed on 38 subjects, all with hearing loss and over 19 years old (20.2%). The mean values of the r-SSQ12-A scores did not change significantly from a statistical point of view (Table 5).

**Figure 4:**
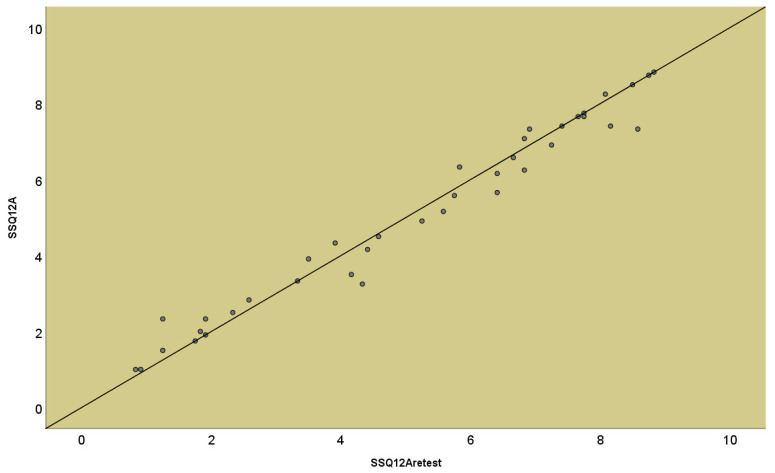
Reliability r-SSQ12A-test/r-SSQ12A-retest. The internal consistency was evaluated by calculating Cronbach’s alpha coefficient, which was 0.976 for the r-SSQ12-A total score with a lambda2 value of 0.9. For the r-SSQ12-B and r-SSQ12-C variants, Cronbach’s alpha coefficient was 0.846 and 0.942, respectively.

The reliability of the scale was confirmed via testing test/retest reliability; ICC = 0.992 (Figure 4).

## 4. Discussion

The perceived impact of hearing loss varies considerably among the individuals affected due to the heterogeneous types of hearing loss, the diverse etiologies of hearing loss, and the different rehabilitation possibilities. Often, people with hearing loss, especially those who rely on an amplification device on the better ear, experience disabilities: poor sound localization in space, poor speech discrimination in noisy environments, and a reduced quality of life. The SSQ12 questionnaire, composed of five items on speech understanding, three on the spatial domain, and four on the qualities of hearing, can explore these deficits. In the field of speech understanding, all scenarios presented in the five questions are marked by the presence of competing noise. Listening to target speech (of interest) together with other direct speech, listening to speech as background noise, and listening to speech in noise are more difficult tasks than hearing and understanding speech in silence [6]. For the spatial domain, one question describes the situation of hearing and identifying a sound source in the external environment, and the other spatial scenarios put the subject in the situation of identifying their position in relation to a moving noise source. The qualities of auditory experience include the ease of listening, listening effort, and the naturalness, clarity, and segregation of different sounds in everyday life [2,3]. The results captured by this variant show that the chosen questions measure the most difficult listening tasks both for a subject with normal hearing and especially for a person with hearing loss.

Sommers (2011) [22] found that the percentage of people with normal audiometric thresholds who self-reported hearing impairment was 12.0%. Kamerer (2022) [23] evaluated 111 adults (aged 19–74 years) with clinically normal hearing (audiometric thresholds ≤ 25 dB HL at frequencies between 0.25 and 8 kHz and bilaterally symmetrical hearing) and reported the mean SSQ12 for subjects with audiometric thresholds ≤ 25 dB HL who rated themselves as having good and very good hearing at 9.5 and lower values in those with self-reported hearing loss. A history of noise exposure and self-reported hearing loss predicted scale scores with good specificity were observed [17,18]. On this basis, we included subjects with similar thresholds who reported very good hearing in the group with normal hearing. 

An r-SSQ12 score of 9.177 ± 0.60 for the group with normal hearing can therefore be explained by the construction of the group (most of the subjects were medical staff of the clinic with above-average education), and is comparable to that for young Turkish (7.8–9.5 NH) [24] and French (8.5–9.54) [25,26] adults. 

Adolescent r-SSQ12 means are comparable to those of other French [27], Italian (8.5–9.5 for normal-hearing subjects and 6.5–8.3 subjects with hearing loss) [28], and Dutch adolescents (8–9.2 for normal-hearing subjects and 3.8–7.9 for subjects with hearing loss) [29].

The mean SSQ score for the group of people with hearing loss, at a mean age of 60, and with sensorineural hearing loss was 5.1 (SD = 1.2, range: 3.17–6.27) for the Iranian scale [30]. The score for a population of Turkish adults with sensorineural hearing loss ranged from 3.9 to 7 [14], and the questionnaire for French adults returned an average of 6.6 [24].

The median r-SSQ12 score for the normal hearing group differs with statistical significance for the total score and for each item according to hearing symmetry. The group with normal hearing had the lowest scores on the speech subscale, as did the BHL group, while the UHL and AHL groups scored lower on the spatial subscale. Subjects with unilateral sensorineural hearing loss had lower scores compared to those in the group with mild and moderate bilateral hearing loss. 

Cronbach’s alpha value is good and close to that of the variants for Turkish Arabic, Iranian, and Spanish [24,31,32]. Cañete recommended using the SSQ12 as a screening tool for hearing loss, with a score of ≤8.5 points for the total average as a cut-off point.

This study has some limitations. It was carried out in a single center (ENT clinic of the Clinical Recovery Hospital). However, this center is representative of subjects with hearing loss considering that it serves the population of North-East Romania in addition to other populations (some subjects were from the Republic of Moldova or from the rest of the country). Our center is the only one using cochlear implants or bone conduction in this area. It deals with the pathology of both children and adults regarding the diagnosis, treatment, and follow-up of these patients. The selection of subjects for this study and for future ones is broad, as the center offers both audiological and surgical services.

Validation focused on subjects with asymmetric loss, and groups of normal subjects or with bilateral hearing loss were constructed that were equivalent in size from volunteers with normal hearing or regular clinic patients. No test of the matching of sample size was performed. We consider that the number of subjects with asymmetric loss or those with profound bilateral hearing loss (who completed the SSQ-C form) is somewhat representative of the Romanian population.

In a future stage, we will analyze the results returned on the SSQ12B and SSQ12C forms at the global and subscale levels. Also, the study shows how to capture the results of rehabilitation with implantable devices for different categories of patients with hearing loss (patients with single-sided deafness or asymmetric deafness and children with congenital or acquired unilateral hearing loss or progressive deafness). The spatial scale and how it alters the perception of hearing quality will be our focus. With all the limitations described, the study can be a starting point for exploring the benefits of rehabilitation interventions, even if it can be used as a screening tool.

## 5. Conclusions

Good repeatability and test–retest correlations are close to those reported by similar studies, and the questionnaire demonstrated its discriminating power between subjects with normal hearing and subjects with hearing loss. Subscale analyses of data from subjects enrolled in studies of rehabilitation with various implantable devices may reveal the perceived impairment of people with hearing loss and the effectiveness of these auditory rehabilitation interventions from the user’s perspective.

## Figures and Tables

**Table 1:** Demographic characteristics in different subgroups with and without hearing loss.

Hearing Loss	Type	Gender		(n)	Mean PTA4 dB	Std. Deviation
Mild and moderate	SNHL	F	worse ear	12	43.75	9.32
			better ear	12	37.5	8.66
		M	worse ear	6	38.33	11.25
			better ear	6	35	12.65
	Mixed	F	worse ear	3	56.66	10.41
			better ear	3	51.67	10.41
	Conductive	F	worse ear	12	49.58	11.17
			better ear	12	24.58	16.44
		M	worse ear	10	48	8.56
			better ear	10	29	14.49
normal hearing	NH	F	worse ear	39	13.84	5.436
			better ear	39	11.54	5.15
		M	worse ear	23	13.261	4.67
			better ear	23	10.87	5.77
Severe and profound	SNHL	F	worse ear	42	98.45	16.39
			better ear	42	48.45	31.46
		M	worse ear	27	102.41	15.65
			better ear	27	40	31.86
	Mixed	F	worse ear	8	80.62	9.04
			better ear	8	49.38	18.41
	Conductive	F	worse ear	1	70	.
			better ear	1	55	.

**Table 2:** Age group and degree of hearing loss in the total study group.

Age Group (years)	Normal Hearing (n = 62)		Mild/Moderate (n = 43)		Severe/Profound (n = 78)		Total (n = 183)	
	n	%	n	%	N	%	n	%
11–19	11	36.7	6	20.0	13	43.3	30	16.4
20–29	12	54.5	1	4.5	9	40.9	22	12.0
30–39	13	48.1	3	11.1	11	40.7	27	14.8
40–49	13	38.2	13	38.2	8	23.5	34	18.6
50–59	13	31.7	5	12.2	23	56.1	41	22.4
60–69	0	0.0	9	52.9	8	47.1	17	9.3
70–79	0	0.0	6	50.0	6	50.0	12	6.6

**Table 3:** Multiple comparisons across types of hearing loss groups.

		Std. Error	*p*	95% Confidence Interval	
				Lower Bound	Upper Bound
NH	SNHL	0.297	0.000	3.4747	5.0620
	conductive	0.432	0.000	0.9891	3.2977
	mixt	0.579	0.000	2.6902	5.7839
SNHL	NH	0.297	0.000	−5.0620	−3.4747
	conductive	0.417	0.000	−3.2390	−1.0108
	mixt	0.568	1.000	−1.5484	1.4858
conductive	NH	0.432	0.000	−3.2977	−0.9891
	SNHL	0.417	0.000	1.0108	3.2390
	mixt	0.649	0.009	0.3604	3.8269
mixt	NH	0.579	0.000	−5.7839	−2.6902
	SNHL	0.568	1.000	−1.4858	1.5484
	conductive	0.649	0.009	−3.8269	−0.3604

**Table 4:** Mean scores on each item on subscales depending on the symmetry of the hearing loss.

Item	NH	UHL	AHL	BHL	F_ANOVA_ Test; *p*
Q12A1	9.58 ± 0.69	6.83 ± 2.37	5.36 ± 2.06	5.22 ± 2.46	0.001
Q12A2	8.65 ± 1.31	5.17 ± 2.58	2.91 ± 2.63	3.73 ± 2.55	0.001
Q12A3	8.81 ± 1.28	5.74 ± 2.60	3.09 ± 2.12	3.70 ± 2.68	0.001
Q12A4	8.69 ± 1.28	4.94 ± 2.57	3.73 ± 3.17	3.49 ± 2.68	0.001
Q12A5	8.92 ± 1.23	5.77 ± 2.75	4.27 ± 3.10	3.92 ± 2.65	0.001
Q12A6	9.42 ± 0.86	4.96 ± 3.12	4.64 ± 2.91	5.60 ± 3.05	0.001
Q12A7	9.37 ± 0.93	5.34 ± 2.83	3.36 ± 2.38	5.24 ± 2.83	0.001
Q12A8	9.18 ± 1.06	5.83 ± 2.60	4.27 ± 3.64	5.19 ± 2.99	0.001
Q12A9	9.34 ± 0.94	6.43 ± 2.51	4.82 ± 3.89	5.19 ± 2.89	0.001
Q12A10	9.37 ± 0.87	7.55 ± 2.41	5.00 ± 3.00	5.32 ± 3.30	0.001
Q12A11	9.35 ± 0.87	7.47 ± 2.22	5.55 ± 2.34	5.59 ± 3.09	0.001
Q12A12	9.44 ± 0.76	6.36 ± 2.48	3.64 ± 2.06	4.75 ± 3.08	0.001

**Table 5:** The development of the mean values of the r-SSQ12-A total score upon retesting.

Item	Test	Retest	Paired Samples T Test
Speech Comprehension Scale			
Q12A1	5.51 ± 2.58	5.56 ± 2.47	0.710
Q12A2	4.41 ± 2.80	4.41 ± 2.73	1.000
Q12A3	4.32 ± 2.81	4.15 ± 2.67	0.392
Q12A4	3.93 ± 2.87	4.22 ± 2.79	0.110
Q12A5	4.44 ± 3.01	4.61 ± 2.91	0.377
Spatial scale			
Q12A6	5.02 ± 3.17	4.85 ± 3.21	0.302
Q12A7	5.12 ± 2.94	5.05 ± 2.94	0.674
Q12A8	4.78 ± 2.75	5.12 ± 2.93	0.075
Hearing quality			
Q12A9	5.59 ± 2.79	5.83 ± 2.50	0.398
Q12A10	6.37 ± 2.83	6.02 ± 2.94	0.147
Q12A11	6.51 ± 2.50	6.17 ± 2.92	0.109
Q12A12	5.24 ± 2.95	5.24 ± 2.80	0.981
Q12A TOTAL	5.09 ± 2.44	5.15 ± 2.58	0.408

## Data Availability

Data are available from authors at reasonable request.

## Supplementary Materials

The following are available online at https://www.mdpi.com/article/10.3390/jpm14010090/s1. Supplementary Materials.
