# Supplementary material for: The Validation of the Speech, Spatial and Qualities of Hearing Scale SSQ12 for Native Romanian Speakers with and without Hearing Impairment
Source: J Pers Med. 2024 Jan 13;14(1):90. doi: 10.3390/jpm14010090 (PMC10821130; doi:10.3390/jpm14010090)
Supplement: Supplementary file 1 [file jpm-14-00090-s001.zip › sup-confirmed/SSQ 12-A romanian (1).pdf]

SSQ12A

Instrucțiuni de completare

Întrebările următoare se referă la abilitatea și experiența dumneavoastră de a auzi și asculta în diferite situații zilnice obișnuite.

Chestionarul se completează încercuind cifra pe care o considerați potrivită pentru experiența proprie în situația prezentată. Fiecare întrebare este insoțită de o scala de la unu la zece. Alegând cifra zece înseamnă că sunteți **perfect** capabil să faceți ceea ce este descris în fiecare scenariu. Dacă încercuiți cifra 0 înseamnă că nu puteți **deloc** să îndepliniți ce este descris.

De exemplu, întrebarea 1 se referă la o conversație pe care o aveți cu o altă persoană în timp ce televizorul este pornit. Dacă înțelegeți conversația în timp ce se aude și programul TV încercuiți cifra 10 din partea dreapta a scalei. Dacă nu înțelegeți decât jumatate din conversația cu persoana din camera încercuiți cifra 5 de la mijlocul scalei și așa mai departe, dacă nu puteți înțelege deloc conversația încercuiți cifra 0 de la capătul din stânga al scalei. Dacă întrebarea nu descrie o situație din viața de zi cu zi care să vi se potivească, încercuiți **NU SE APLICĂ**. Dacă doriți puteți explica pe rândul corespunzător **NU SE APLICĂ** de ce ați ales acest răspuns.

Nume Prenume

Vârsta actuală

Data completării

**Vă rugăm să alegeți o variantă punând un X în dreptul propoziției potrivite.**	
**Nu am proteză auditivă.**	☐
**Am proteză auditivă la urechea dreaptă.**	☐
**Am proteză auditivă la urechea stângă.**	☐
**Am proteze auditive la ambele urechi**	☐

Dacă aveți proteze sau ajutor pentru auz de cât timp le folosiți?	
**Urechea dreaptă**	luni
**Tip ajutor**	ani
**Urechea stângă**	luni
**Tip ajutor**	ani

1. Vorbești cu cineva într-o cameră unde este un televizor deschis. Fără a închide televizorul poți înțelege ce spune persoana cu care vorbești?

deloc *perfect*

0 1 2 3 4 5 6 7 8 9 10

Nu se aplică ☐ ……………………………………………………………………………

2. Asculți ce spune cineva care vorbește cu tine și în același timp încerci să urmărești știrile la televizor. Înțelegi ce spune acea persoană și ce se spune la TV?

deloc *perfect*

0 1 2 3 4 5 6 7 8 9 10

Nu se aplică ☐ ……………………………………………………………………………

3. Discuți cu cineva într-o cameră în care multe alte persoane vorbesc. Înțelegi ce îți spune acea persoană ?

deloc *perfect*

0 1 2 3 4 5 6 7 8 9 10

Nu se aplică ☐ ……………………………………………………………………………

4.Ești într-un grup de aproximativ cinci persoane într-un restaurant aglomerat. Îi poți vedea pe toți din grup. Înțelegi ce se discută?

deloc *perfect*

0 1 2 3 4 5 6 7 8 9 10

Nu se aplică ☐ ....

5. Ești cu un grup și conversația se mută de la o persoană la alta. Poți urmări ușor discuția fără a pierde începutul a ceea ce spune fiecare nou vorbitor?

deloc *perfect*

0 1 2 3 4 5 6 7 8 9 10

Nu se aplică ☐ ....

6. Ești afară și un câine latră tare. Poți spune imediat unde este fără să te uiți?

deloc *perfect*

0 1 2 3 4 5 6 7 8 9 10 Nu se aplică ☐ ....

7. Poți spune cât de departe este un autobuz sau un camion după sunetul pe care îl auzi?

deloc *perfect*

0 1 2 3 4 5 6 7 8 9 10

Nu se aplică ☐ ……………………………………………………………………

8. Poți spune dacă un autobuz sau un camion se apropie sau se îndepărtează după sunetul pe care îl auzi?

deloc *perfect*

0 1 2 3 4 5 6 7 8 9 10

Nu se aplică ☐ ……………………………………………………………………

9. Când auzi mai mult de un sunet odată ai impresia că se aude ca unul singur amestecat?

amestecat *neamestecat*

0 1 2 3 4 5 6 7 8 9 10

Nu se aplică ☐ ……………………………………………………………………

10. Când asculți muzică îți dai seama la ce instrument se cântă?

deloc *perfect*

0 1 2 3 4 5 6 7 8 9 10

Nu se aplică ☐ ……………………………………………………………………………….

11. Sunetele de zi cu zi pe care le auzi cu ușurință par clare(nu înfundate)?

deloc *perfect*

0 1 2 3 4 5 6 7 8 9 10

Nu se aplică ☐ ……………………………………………………………………

12. Trebuie să te concentrezi foarte tare pentru a înțelege ceva sau pe cineva?

Foarte tare *nu e necesar*

0 1 2 3 4 5 6 7 8 9 10

Nu se aplică ☐ ……………………………………………………………

Chestionar dizabilitate administrat împreună cu SSQ

Instrucțiuni de completare

Următoarele întrebări se referă la trăirile pe care le aveți referitoare la starea auzului. Puteți alege o singură variantă de răspuns din cele cinci pe care o veți nota pe rândul corespunzător fiecărei întrebări.

Alternative de răspuns

(A) Niciodată

(B) Rareori

(C) Uneori

(D) Adesea

(E) Aproape mereu

1. Cât de des dificultatea dumneavoastră de auz limitează lucrurile pe care le faceți?

2. Cât de des vă simțiți îngrijorat sau anxios din cauza dificultatii de auz?

3. Ca urmare a dificultății de auz cât de des resimțiți jenă în compania altor oameni?

4. Cât de des vă este afectată încrederea în sine de dificultatea de auz?

5. Cât de des vă face dificultatea de auz să vă simțiți nervos sau inconfortabil ?

6. Cât de des conștientizați problema de auz?

7. Cât de des afectează problemele cu auzul felul in care vă simțiți?

8. Cât de des sunteți deranjat de dificulatea de auz?

9. Cât de des vă simțiți înclinat să evitați situațiile sociale din cauza dificultății de auz?

10. Cât de des vă simțiți rupt de lucruri din cauza dificultații de auz?

11. Cât de des dificultatea de auz vă limitează interacțiunile sociale sau viața personală?

12. Cât de des vă simțiți încordat și obosit din cauza dificultății de auz?

MUȚUMESC.

SSQ12-A
