# Supplementary material for: The Validation of the Speech, Spatial and Qualities of Hearing Scale SSQ12 for Native Romanian Speakers with and without Hearing Impairment
Source: J Pers Med. 2024 Jan 13;14(1):90. doi: 10.3390/jpm14010090 (PMC10821130; doi:10.3390/jpm14010090)
Supplement: Supplementary file 1 [file jpm-14-00090-s001.zip › sup-confirmed/SSQ 12-B romanian.pdf]

Următoarele întrebări se referă la abilitatea și experiența dumneavoastră referitoare la auz în diferite situații obișnuite. Ați răspuns la aceste întrebări înainte de a primi dispozitivul sau dispozitivele auditive.

Am dori să răspundeți la aceleași întrebări, dar comparând capacitatea și experiența dumneavostră acum, folosind aparatul sau aparatele auditive cu abilitățile și experiența dumneavoastră fără să fiți echipați cu ajutor auditiv.

Pentru fiecare întrebare, puneți un semn, de exemplu un X, oriunde pe scala care variază de la -5 la 0 la +5, afișată pentru fiecare întrebare.

• Dacă vă descurcați mai bine acum, cu aparatul/aparatele auditive actuale comparativ cu situația precedentă în situaţia descrisă de o anume întrebare puneți semnul în dreapta lui 0.

• Dacă vă descurcați mai rău, atunci puneți semnul la stânga lui 0.

• Dacă lucrurile nu sunt diferite, puneți semnul în jurul valorii de 0.

• Dacă lucrurile stau mult mai rău, marcați în jurul valorii de -5; dacă lucrurile sunt mult mai bune marcați-l în jurul valorii de +5.

Ne așteptăm ca toate întrebările să fie relevante pentru experiența zilnică dar dacă o întrebare descrie o situație care nu vi se aplică marcați căsuța „Nu se aplică”. Dacă este posibil vă rugăm să explicați de ce nu se aplică în cazul dvs.

Numele dumneavoastră :   Data de azi   Vârsta

**Vă rugăm să alegeți o variantă punând un X în dreptul propoziției potrivite.**	
**Nu am proteză auditivă.**	☐
**Am proteză auditivă la urechea dreaptă.**	☐
**Am proteză auditivă la urechea stângă.**	☐
**Am proteze auditive la ambele urechi**	☐

Dacă folosiți proteze sau ajutor pentru auz puteți să ne spuneți de cât timp?	
**Urechea dreaptă**	luni
**Tip ajutor**	ani
	luni
**Urechea stângă**	
**Tip ajutor**	ani

1. Vorbești cu o persoană într-o cameră unde este un televizor deschis. Fără a închide televizorul poți înțelege ce spune persoana cu care vorbești?

comparând abilitatea actuală cu cea de dinainte de a primi aparatul/aparatele auditiv(e)

mult mai rea *nici o schimbare* *mult mai bună*

-5 -4 -3 -2 -1 0 +1 +2 +3 +4 +5

Nu se aplică ☐ ....

2. Asculți ce spune cineva care vorbește cu tine și în același timp încerci să urmărești știrile la televizor. Înțelegi ce spune acea persoană și ce se spune la TV?

comparând abilitatea actuală cu cea de dinainte de a primi aparatul/aparatele auditiv(e)

mult mai rea *nici o schimbare* *mult mai bună*

-5 -4 -3 -2 -1 0 +1 +2 +3 +4 +5

Nu se aplică ☐ ....

3. Discuți cu cineva într-o cameră în care multe alte persoane vorbesc. Înțelegi ce îți spune acea persoană?

comparând abilitatea actuală cu cea de dinainte de a primi aparatul/aparatele auditiv(e)

mult mai rea *nici o schimbare* *mult mai bună*

-5 -4 -3 -2 -1 0 +1 +2 +3 +4 +5

Nu se aplică ☐ ....

4.Ești într-un grup de aproximativ cinci persoane într-un restaurant aglomerat. Îi poți vedea pe toți din grup. Înțelegi ce se discută?

comparând abilitatea actuală cu cea de dinainte de a primi aparatul/aparatele auditiv(e)

mult mai rea *nici o schimbare* *mult mai bună*

-5 -4 -3 -2 -1 0 +1 +2 +3 +4 +5

Nu se aplică ☐ ....

5. Ești cu un grup și conversația se mută de la o persoană la alta. Poți urmări ușor discuția fără a pierde începutul a ceea ce spune fiecare nou vorbitor?

comparând abilitatea actuală cu cea de dinainte de a primi aparatul/aparatele auditiv(e)

mult mai rea *nici o schimbare* *mult mai bună*

-5 -4 -3 -2 -1 0 +1 +2 +3 +4 +5

Nu se aplică ☐ ....

6. Ești afară și un câine latră tare. Poți spune imediat unde este fără să te uiți?

comparând abilitatea actuală cu cea de dinainte de a primi aparatul/aparatele auditiv(e)

mult mai rea *nici o schimbare* *mult mai bună*

-5 -4 -3 -2 -1 0 +1 +2 +3 +4 +5

Nu se aplică ☐ ....

7. Poți spune cât de departe este un autobuz sau un camion după sunetul pe care îl auzi?

comparând abilitatea actuală cu cea de dinainte de a primi aparatul/aparatele auditiv(e)

mult mai rea *nici o schimbare* *mult mai bună*

-5 -4 -3 -2 -1 0 +1 +2 +3 +4 +5

Nu se aplică ☐ ....

8. Poți spune dacă un autobuz sau un camion se apropie sau se îndepărtează după sunetul pe care îl auzi?

comparând abilitatea actuală cu cea de dinainte de a primi aparatul/aparatele auditiv(e)

mult mai rea *nici o schimbare* *mult mai bună*

-5 -4 -3 -2 -1 0 +1 +2 +3 +4 +5

Nu se aplică ☐ ....

9. Când auzi mai mult de un sunet odată ai impresia că se aude ca un singur sunet amestecat?

comparând abilitatea actuală cu cea de dinainte de a primi aparatul/aparatele auditiv(e)

mult mai rea *nici o schimbare* *mult mai bună*

-5 -4 -3 -2 -1 0 +1 +2 +3 +4 +5

Nu se aplică ☐ ....

10. Când asculți muzică îți dai seama la ce instrument se cântă?

comparând abilitatea actuală cu cea de dinainte de a primi aparatul/aparatele auditiv(e)

mult mai rea *nici o schimbare* *mult mai bună*

-5 -4 -3 -2 -1 0 +1 +2 +3 +4 +5

Nu se aplică ☐ ....

11. Sunetele de zi cu zi pe care le auzi cu ușurință par clare(nu înfundate)?

comparând abilitatea actuală cu cea de dinainte de a primi aparatul/aparatele auditiv(e)

mult mai rea *nici o schimbare* *mult mai bună*

-5 -4 -3 -2 -1 0 +1 +2 +3 +4 +5

Nu se aplică ☐ ....

12. Trebuie să te concentrezi foarte tare pentru a înțelege ceva sau pe cineva?

comparând abilitatea actuală cu cea de dinainte de a primi aparatul/aparatele auditiv(e)

mult mai rea *nici o schimbare* *mult mai bună*

-5 -4 -3 -2 -1 0 +1 +2 +3 +4 +5

Nu se aplică ☐ ....

Chestionar dizabilitate administrat împreună cu SSQ

Instrucțiuni de completare

Următoarele întrebări se referă la trăirile pe care le aveți referitoare la starea auzului. Puteți alege o singură variantă de răspuns din cele cinci pe care o veți nota pe rândul corespunzător fiecărei întrebări.

Alternative de răspuns

(A) Niciodată

(B) Rareori

(C) Uneori

(D) Adesea

(E) Aproape mereu

1. Cât de des dificultatea dumneavoastră de auz limitează lucrurile pe care le faceți?

2. Cât de des vă simțiți îngrijorat sau anxios din cauza dificultatii de auz?

3. Ca urmare a dificultății de auz cât de des resimțiți jenă în compania altor oameni?

4. Cât de des vă este afectată încrederea în sine de dificultatea   de auz?

5. Cât de des vă face dificultatea de auz să vă simțiți nervos sau inconfortabil ?

6. Cât de des conștientizați problema de auz?

7. Cât de des afectează problemele cu auzul felul in care vă simțiți?

8. Cât de des sunteți deranjat de dificulatea de auz?

9. Cât de des vă simțiți înclinat să evitați situațiile sociale din cauza dificultății de auz?

10. Cât de des vă simțiți rupt de lucruri din cauza dificultații de auz?

11. Cât de des dificultatea de auz vă limitează interacțiunile sociale sau viața personală?

12. Cât de des vă simțiți încordat și obosit din cauza dificultății de auz?

MUȚUMESC.

SSQ12-B
